# Evidence for Enhanced Exosome Production in Aromatase Inhibitor-Resistant Breast Cancer Cells

**DOI:** 10.3390/ijms21165841

**Published:** 2020-08-14

**Authors:** Giuseppina Augimeri, Giusi La Camera, Luca Gelsomino, Cinzia Giordano, Salvatore Panza, Diego Sisci, Catia Morelli, Balázs Győrffy, Daniela Bonofiglio, Sebastiano Andò, Ines Barone, Stefania Catalano

**Affiliations:** 1 Department of Pharmacy, Health and Nutritional Sciences, Via P Bucci, University of Calabria, 87036 Arcavacata di Rende (CS), Italy; giusy.augimeri@gmail.com (G.A.); giusylacamera93@gmail.com (G.L.C.); luca.gelsomino@unical.it (L.G.); cinzia.giordano@unical.it (C.G.); sasapanza@libero.it (S.P.); dsisci@unical.it (D.S.); catia.morelli@unical.it (C.M.); daniela.bonofiglio@unical.it (D.B.); sebastiano.ando@unical.it (S.A.); 2 Centro Sanitario, Via P Bucci, University of Calabria, 87036 Arcavacata di Rende (CS), Italy; 3 Bioinformatics and 2nd Department of Pediatrics, Semmelweis University, 1094 Budapest, Hungary; zsalab2@yahoo.com; 4 TTK Cancer Biomarker Research Group, 1117 Budapest, Hungary

**Keywords:** breast cancer, endocrine resistance, exosomes, Rab GTPases

## Abstract

Aromatase inhibitors (AIs) represent the standard anti-hormonal therapy for post-menopausal estrogen receptor-positive breast cancer, but their efficacy is limited by the emergence of AI resistance (AI^R^). Exosomes act as vehicles to engender cancer progression and drug resistance. The goal of this work was to study exosome contribution in AI^R^ mechanisms, using estrogen-dependent MCF-7 breast cancer cells as models and MCF-7 LTED (Long-Term Estrogen Deprived) subline, modeling AI^R^. We found that exosome secretion was significantly increased in MCF-7 LTED cells compared to MCF-7 cells. MCF-7 LTED cells also exhibited a higher amount of exosomal RNA and proteins than MCF-7 cells. Proteomic analysis revealed significant alterations in the cellular proteome. Indeed, we showed an enrichment of proteins frequently identified in exosomes in MCF-7 LTED cells. The most up-regulated proteins in MCF-7 LTED cells were represented by Rab GTPases, important vesicle transport-regulators in cancer, that are significantly mapped in “small GTPase-mediated signal transduction”, “protein transport” and “vesicle-mediated transport” Gene Ontology categories. Expression of selected Rab GTPases was validated by immunoblotting. Collectively, we evidence, for the first time, that AI^R^ breast cancer cells display an increased capability to release exosomes, which may be associated with an enhanced Rab GTPase expression. These data provide the rationale for further studies directed at clarifying exosome’s role on endocrine therapy, with the aim to offer relevant markers and druggable therapeutic targets for the management of hormone-resistant breast cancers.

## 1. Introduction

According to the GLOBOCAN statistics in 2018, breast cancer represents the most commonly diagnosed cancer and the deadliest type of malignancy among the female population on a world scale, showing morbidity and mortality rates of ~25% and ~15%, respectively [1]. Clinical decisions are generally dependent on disease stage and expression of estrogen (ER) and progesterone (PR) receptors, epidermal growth factor 2 receptor (HER2) and Ki-67. Hormone receptor-positive breast carcinomas account for almost 70–80% of all cancer cases and mainly overlap with luminal molecular subtypes [2]. In these tumors, endocrine-targeted treatments using aromatase inhibitors (AIs, i.e., letrozole, anastrozole and exemestane) represent the mainstay of the standard care both in the adjuvant and recurrent settings. Unfortunately, the development of “de novo” or acquired resistance to a prolonged estrogen withdrawal profoundly affects patient prognosis, which is a significant global concern [3]. To date, the hallmarks of hormonal resistance were studied thoroughly and may include the loss or mutations of ER, activation of growth factor signaling-dependent pathways, alterations of crucial cell cycle checkpoints, induction of epithelial-to-mesenchymal transition (EMT), cancer stem cell activity, and heterotypic cellular interactions within the tumour microenvironment [4,5,6,7,8,9,10,11]. However, despite major advances in our understanding of these molecular events, we are still unable to effectively treat hormone-resistant diseases, highlighting the need to explore novel, clinically relevant markers and therapeutic targets.

Exosomes are nanoscale membrane vesicles with a diameter of 20–200 nm, belonging to the large family of the small extracellular vesicles (EVs), which are generated in the cells and released into the extracellular space. Although current separation and characterization methods for the EVs do not really allow us to define the specific subclass of EVs such as exosomes, as described in the “Minimal information for studies of extracellular vesicles 2018-(MISE2018)” [12], a large amount of literature in recent decades has highlighted the involvement of these small EVs in cancer biology. The attracted considerable interest in these vesicles relies on their ability to shuttle from one cell to another and deliver signals or messages to particular recipient cells. Indeed, they contain several biomolecules, such as nucleic acids, proteins, and lipids, that mediate crosstalk between neighboring or anatomically distant cells [13]. In recent years, it has been reported that the mechanisms of exosome biogenesis/release are deregulated in cancer, including breast cancer, with increased exosome counts in cancer cell lines and in patients’ blood [14,15]. Indeed, breast cancer-derived exosomes, on the basis of their own cargo, may exhibit a wide array of biological activities, such as the induction of reactive oxygen species, autophagy and DNA damage repair response in normal human primary mammary epithelial cells [16], oncogenic transformation and tumor formation [17,18,19], impairment of the immune response [20,21], stimulation of mesenchymal stem cell differentiation into cancer-associated fibroblasts (CAFs) [22] along with increased fibroblast proliferation and lifespan [23]. On the other hand, only a few studies demonstrated the effect of the horizontal transfer of the resistance between cancer cells. For instance, tumor exosomes might contribute to chemotherapy failure by transferring functional p-glycoprotein from chemo-resistant MCF-7 cells [24], or interfere with the antineoplastic properties of Trastuzumab through different mechanisms [25,26,27]. Overexpression of the thymidine kinase 1 (TK1) and the cyclin-dependent kinase 9 (CDK9) in plasma-derived exosomes was significantly correlated with clinical resistance to CDK4/6 inhibitors in metastatic breast cancer patients [28]. In addition, exosomes from tamoxifen-resistant MCF-7 cells, by transfer miR-221/222 [29] or lncRNA UCA1 (Urothelial Carcinoma-Associated 1) [30], promoted the tamoxifen-resistant phenotype in MCF-7-sensitive cells. It has also been found that exosomes from a tamoxifen-resistant subline led to the irreversible cross-resistance of the parental MCF-7 cells to both tamoxifen and the anti-diabetic drug metformin [31]. Interestingly, the horizontal transfer of mitochondrial DNA from exosomes promoted an escape from dormancy in tamoxifen-resistant breast cancer cell lines [32]. Although it is becoming widely acknowledged to the scientific community the tumor-supportive role of the intercellular communications mediated by the secretion of exosomes from cancer cells, their involvement in the mechanisms underlying insensitivity to AI treatments has not been yet researched. In the present study, we show that resistance to AIs is associated to an enhanced exosome production, which appears to be related to an increased Rab GTPase expression.

## 2. Results

### 2.1. MCF-7 LTED (Long-Term Estrogen Deprived) Cells Exhibit Increased Exosome Production

**Figure 1:**
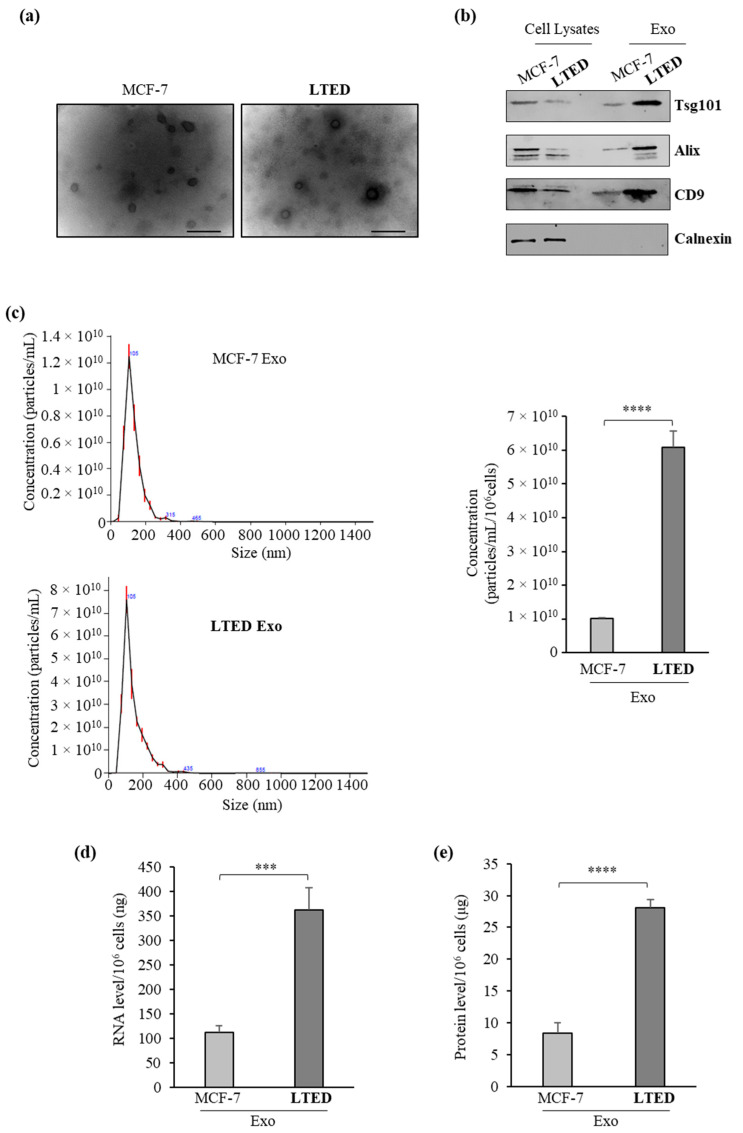
Increased release of exosomes from MCF-7 LTED cells compared to MCF-7 cells. (**a**) Representative micrograph of transmission electron microscopy (TEM) of exosomes from conditioned medium of MCF-7 (MCF-7 Exo) and MCF-7 LTED (LTED Exo) breast cancer cells. Scale bar, 100 nm; (**b**) Immunoblotting showing expression of the exosome hallmarks Tsg101, Alix and CD9 in equal amount (4 µg) of exosome lysates (Exo) and whole cell lysates of MCF-7 and MCF-7 LTED cells. Calnexin was used to ensure that exosome samples were not contaminated with endoplasmic reticulum proteins; (**c**) Size distribution and concentration profiles of exosomes (Exo) recovered from MCF-7 and MCF-7 LTED breast cancer cell conditioned media (CM), measured by nanoparticle tracking analysis (NTA). The hystogram represents the mean ± S.D. of exosome concentration (particles/mL/10^6^ cells) of 5 analyses; (**d**) Quantitation of average total amount of exosomal RNA per 10^6^ cells; (**e**) Quantitation of average total amount of exosomal proteins per 10^6^ cells. ***, *p* < 0.0005; ****, *p* < 0.0001.

As experimental models to evaluate whether AI^R^ may result in any changes in exosome production, we used parental human ERα-positive MCF-7 breast cancer cells and MCF-7 LTED (Long-Term Estrogen Deprived) cells, which have gradually acquired estrogen independence after six-month culture in estrogen/steroid-free conditions, thereby modeling AI^R^ [33]. In this cell line, well characterized for its resistant phenotype, we observed a low proliferation rate compared to their parental counterpart MCF-7 cells, having a doubling time of 54 h compared to 28 h. First, we isolated exosomes from the conditioned medium of MCF-7 or MCF-7 LTED breast cancer cells cultured in the absence of serum to circumvent the collection of contaminating vesicles from FBS (Fetal Bovine Serum). Exosome isolation was performed by using a well-established ultracentrifugation scheme [34]. The obtained 100,000× *g* pellet that represents the fraction of exosomes was characterized by transmission electron microscopy (TEM), immunoblot analysis and quantitative Nanoparticle Tracking Analysis (NTA). TEM images indicated that isolated vesicles were membrane-encapsulated particles with rounded shaped morphology, characteristic of exosomes (Figure 1a), while the identity of released exosomes was confirmed by the expression of classical exosomal markers, including Tumor susceptibility gene 101 (Tsg101), Alix and CD9 in exosome lysates (Figure 1b). As expected, the expression of the endoplasmic reticulum protein Calnexin was not detected in both samples (Figure 1b). In addition, NTA showed that the average size of exosomes seeded from MCF-7 (MCF-7-Exo) and MCF-7 LTED (LTED-Exo) cells was 127.6 ± 2.9 and 141.7 ± 1.6 nm, respectively, highlighting that the majority of the purified particles were in the expected size range to be defined as exosomes (Figure 1c). Interestingly, when we analyzed the concentration of the secreted vesicles by using NTA, we found that the numbers of the exosomes (particles/mL/10^6^ cells) released in the conditioned medium of MCF-7 LTED cells increased about six-fold compared to those of exosomes isolated from parental cells (6.09 × 10^10^ ± 0.48 × 10^10^ versus 1.01 × 10^10^ ± 1.96 × 10^8^) (Figure 1c). We further determined the concentration of RNA and proteins within the exosomes and found similar results. Indeed, exosomes released from MCF-7 LTED cells exhibited a higher amount of RNA and proteins than those released from MCF-7 cells (Figure 1d,e). Moreover, since also apoptotic cells might increase the release of the extracellular vesicles displaying a broad size range, including exosomes, ectosomes/microvesicles and the larger “apoptotic body” [35], we tested if apoptosis might affect our results. We did not find any signs of apoptosis in MCF-7 and MCF-7 LTED cells, in the same experimental conditions used to obtain the exosome-enriched conditioned media, as demonstrated by the absence of changes in the internucleosomal fragmentation profile of genomic DNA, evaluated by TUNEL assay, and in the proteolysis of poly (ADP-ribose) polymerase (PARP), a known substrate of effector caspases (Supplementary Figure S1). Thus, exosome secretion was significantly increased in MCF-7 LTED cells compared to MCF-7 cells, further indicating that AI^R^ phenotype might be associated with an enhanced capability of breast cancer cells to release exosomes.

### 2.2. Quantitative Proteomic Analysis Shows Extensive Changes of Protein Expression in MCF-7 LTED Cells Compared with MCF-7 Cells

**Figure 2:**
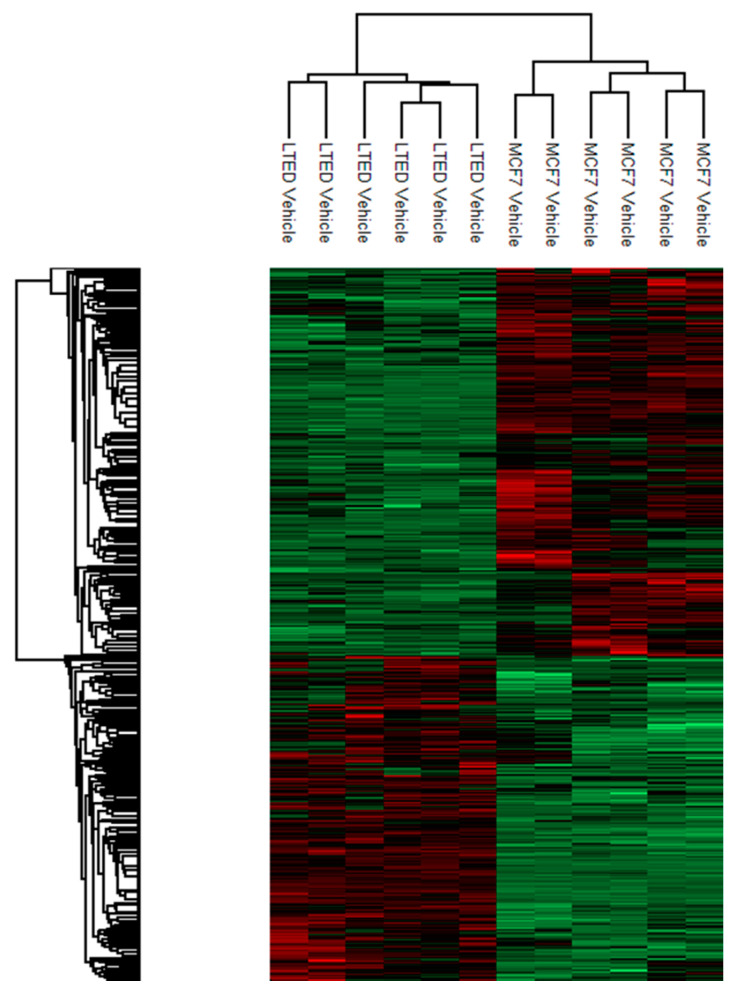
Heatmap representing supervised hierarchical clustering of the differentially expressed proteins in MCF-7 and MCF-7 LTED cells. Heatmap coding uses increasing brightness of red for degree of up-regulation and green for down-regulation. Black color stands for a median expression level.

To gain insight into the underlying molecular mechanisms responsible for the increased production of exosomes in MCF-7 LTED cells, quantitative proteome profiling of MCF-7 and MCF-7 LTED cells was carried out through label-free mass spectrometry using a nano-liquid chromatography coupled to an electrospray ionization-mass spectrometry (nLC-ESI-MS/MS). This comparison allowed the identification of 2794 differentially expressed proteins (FDR < 0.05), of which 811 were significantly down-regulated and 795 significantly up-regulated in MCF-7 cells considering |1.5| fold as a cut-off. Thus, MCF-7 LTED cells have incurred extensive alterations in their proteome. A heatmap to display results of the supervised hierarchical clustering is shown in Figure 2.

These differentially expressed proteins were then subjected to Gene Ontology (GO) analysis to rank enriched biological processes (Table 1). Within this category, we found that that GO terms with the highest enrichment scores were related to “Translation” and “RNA splicing”. Notably, in line with our previous findings, other high-ranking GO terms were related to “protein transport” and “vesicle-mediated transport”.

**Figure 3:**
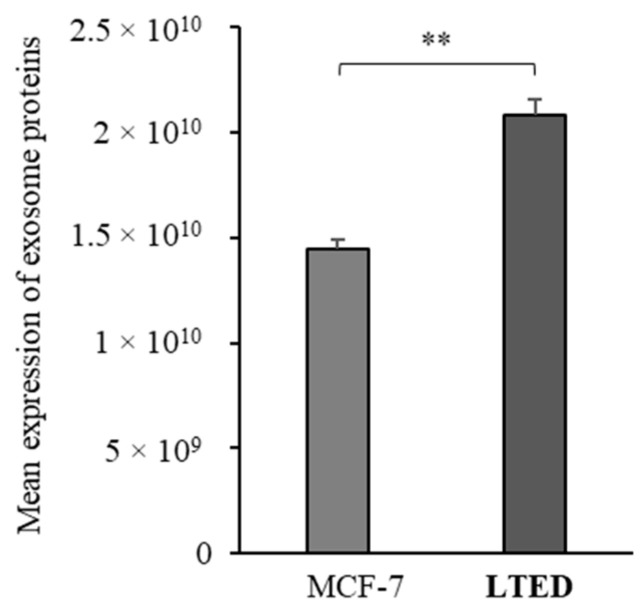
Differential expression of proteins identified in exosomes, revealed by Exocarta, using their mean expression as a signature. **, *p* < 0.005.

In addition, referring to Exocarta (www.exocarta.org), a database for exosomal cargo, more than 50 proteins were detected in the proteomic profiles of both cell lines. Indeed, using the mean expression of all the genes as a signature, a significant enrichment of proteins that are more often identified in exosomes has been observed in MCF-7 LTED cells compared to MCF-7 cells (Figure 3).

### 2.3. MCF-7 LTED Cells Show Enhanced Rab GTPase Protein Expression

Proteomic analysis revealed multiple, functionally distinct proteins that are significantly altered in their expression among the two cell lines. Listed in Table 2 are proteins selected for either their large fold changes and perceived relevance for exosome production. Interestingly, the most up-regulated proteins in MCF-7 LTED cells were represented by Rab GTPases, important vesicle transport regulators in cancer, suggesting that MCF-7 LTED cells exhibited an enrichement of Rab GTPases. As expected, these proteins are significantly mapped in “small GTPase mediated signal transduction”, “protein transport” and “vesicle-mediated transport” GO categories (Fold enrichment = 42.14 and *p* = 2.55 × 10^−29^, Fold enrichment = 16.9 and *p* = 4.83 × 10^−22^, Fold enrichment = 10.6 and *p* = 5.13 × 10^−7^, respectively).

**Figure 4:**
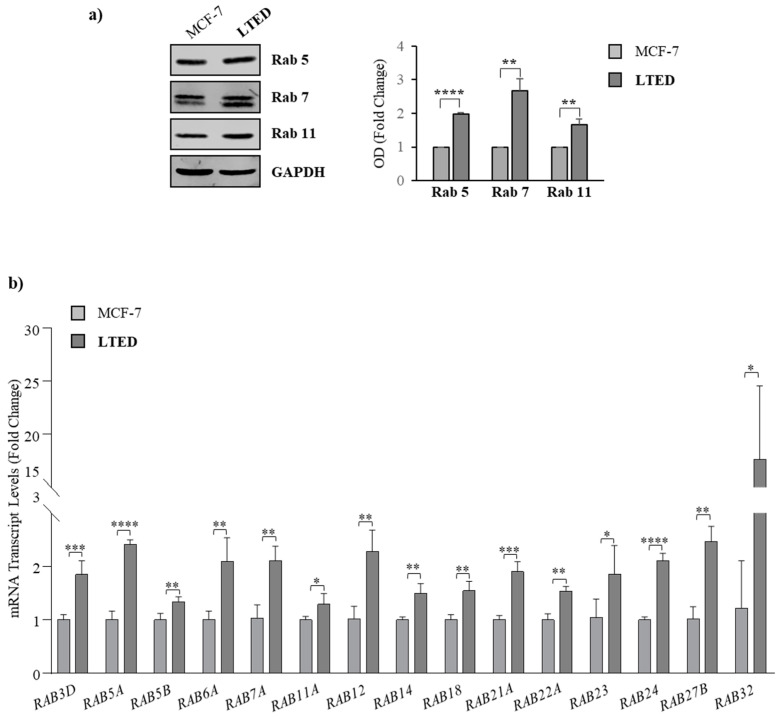
Rab expression in MCF-7 and MCF-7 LTED cells. (**a**) Immunoblot analysis showing protein levels of Rab5, Rab7 and Rab11 in MCF-7 and MCF-7 LTED cell lysates. GAPDH was used as a control for equal loading and transfer. The histogram represents the average fold change ± S.D. of three separate experiments in which band intensities were evaluated in terms of optical density arbitrary units (OD), and expressed as fold change versus MCF-7 cells; (**b**) Real-time RT-PCR for mRNA levels of *RAB* genes in MCF-7 and MCF-7-LTED cells. Data are expressed as means ± S.D. of three different experiments, each performed in triplicate. *, *p* < 0.05; ** *p* < 0.005; ***, *p* < 0.0005; ****, *p* < 0.0001.

To confirm the protein expression profile obtained in our proteomic analysis, we compared the expression of selected Rab GTPases, including Rab5, Rab7 and Rab11, for validation by immunoblotting. Results in Figure 4a showed an increase in the expression levels of Rab5 (approximately two-fold), Rab7 (approximately three-fold) and Rab 11 (approximately two-fold) in MCF-7 LTED cells compared to MCF-7 cells. Additionally, we performed real-time RT-PCR to analyze the mRNA expression levels of Rab GTPases. As shown in the Figure 4b, we found a significant increase in the mRNA levels for all the tested *RAB* genes in MCF-7 LTED cells compared to the parental MCF-7 cells, suggesting the existence of a possible regulation at transcriptional level of the *RAB* gene expression in the AI^R^ cells. Given the important role of Rab GTPases in modulating numerous steps of vesicle trafficking, these results may indicate that the increase in exosome production evidenced in MCF-7 LTED cells may be associated with an enhanced Rab GTPase protein expression. Subsequent studies will be aimed at assessing the role of specific Rab GTPases, the significance of the increased production of exosomes by aromatase inhibitor resistant cells along with any differences in their exosomal content.

## 3. Discussion

Despite significant improvements in the treatment of ER-positive breast cancer following the introduction of AIs, “de novo” and acquired resistance is still an important concern clinically. For this reason, intensive research lines are currently underway and they are aimed at identifying further molecular markers and targets considering alterations in cancer proteomic profiles for more effective personalized therapies. In the present study, we show that resistance to AI treatments is associated to an enhanced exosome production, which appears to be related with an increased Rab GTPase protein expression.

Membrane trafficking machinery is characterized by a multifaceted network of signaling pathways able to connect various membrane-bound organelles of eukaryotic cells. Although each pathway is controlled by a specific set of components, they all enclose Rab GTPases that function as master regulators. Indeed, Rabs can virtually regulate all steps of membrane traffic from the formation of the transport vesicle at the donor membrane to its fusion at the target membrane. Rabs represent the largest family of small Ras-like GTPases with more than 70 members identified in humans that can be classified in several phylogenetic and functional groups [36]. They classically act as molecular switches by cycling between their active (GTP-bound) and inactive (GDP-bound) forms. The GTP-bound state can interact with several structurally and functionally effector proteins that select cargo, facilitate vesicle movement and verify the right site of fusion.

Although, in recent years, advanced progress toward understanding the regulation of exosome biogenesis and secretion by various Rab GTPases has been made, only a few Rab proteins have been shown to play a direct and significant role in these events. Particularly, Rab27A and Rab27B have been reported as the main proteins involved in the regulation of exosome release, while Rab9, Rab5 and Rab2, are generally associated with the endocytic pathway [37]. In addition, Rab11 and Rab35 have been demonstrated to regulate the recycling of membrane components from the endosomal compartment to the plasma membrane having a role in exosome production in different cell types [38]. Interestingly, we found by a quantitative proteomic analysis of the whole cell lysates of MCF-7 LTED (resistant) versus parental (sensitive) cells a significant increase in the expression of Rab GTPase proteins and identified “protein transport” and “vesicle-mediated transport” among the top ten enriched biological processes. Particularly, we found increased levels of Rab27B, Rab5 and Rab11 in terms of protein expression and mRNA content in AI^R^ breast cancer cells. An increased expression of Rab GTPases in our resistant breast cancer cell model well fits with several findings reporting that these important vesicle transport regulators play essential roles in several cancer types [39,40,41,42,43], including breast cancers.

For instance, gene amplification and overexpression of Rab2A promoted breast cancer stem cell expansion via Erk1/2 activation and was associated with poor clinical outcome in patients with breast carcinoma [44]. Rab5A protein expression was correlated to enhanced migration of breast cancer cells and lymphatic dissemination in human breast cancer specimens [45,46]. More recently, a function of Rab7 in the proliferation, invasion, and xenograft tumor development of breast cancer cells was also reported [47], while Rab11, has been demonstrate to contributes to breast cancer cell invasion through trafficking of the α6β4 Integrin [48]. Moreover, Rabs have been found to participate to the intercellular communication between cancerous and stromal cells within the tumor microenvironment [49] and to the development of drug resistance. In this context, Rab27-dependent secretion of exosomes and metalloproteinases resulted into the mobilization of a pro-tumoral neutrophil population, thus supporting growth of a mouse mammary tumor and its lung dissemination [50]. Exosome shuttling between tumor-associated macrophages and cancer cells has been shown to be modulated by Rab27A/B and this mechanism has been involved to chemotherapy resistance [51]. Another Rab protein conveying resistance to a chemotherapeutic drug is the secretory Rab8, through an increased secretion of the cisplatin-resistance-associated protein TMEM205 (transmembrane protein 205) [52]. Moreover, STAT3/Rabs-mediated exosome release was correlated with a more aggressive and chemoresistant cancer phenotype under hypoxic conditions [53] and a recent study demonstrated a role for Rab18 in resistance to cisplatin-induced apoptosis [54].

In AI^R^ breast cancer cells, we found an increased capability to release exosomes as revealed by Nanoparticle Tracking Analysis, which may be associated with the enhanced Rab GTPase expression. Concomitantly, exosomes released from resistant cells exhibited a higher amount of RNA and proteins than those secreted by parental cells. Moreover, based on Exocarta database, we also found in AI^R^ breast cancer cells a significant enrichment of proteins that are often identified in exosomes.

## 4. Materials and Methods

### 4.1. Antibodies

Human anti-RAB5, RAB7, and RAB11 antibodies (Rab Family Antibody Sampler Kit #9385) were acquired from Cell Signaling Technology (Danvers, MA, USA); human anti-GAPDH (sc-47724), anti-Calnexin (sc-11397) and anti-PARP (sc-7150) antibodies were from Santa Cruz Biotechnology (Dallas, TX, USA); human anti-Tsg101 (#MA1-23296) antibody was from Invitrogen (Carlsbad, CA, USA); anti-Alix (ab186429) and anti-CD9 (ab92726) antibodies were acquired from Abcam (Cambridge, UK).

### 4.2. Cell Cultures

Human breast cancer cell line MCF-7 was from American Type Culture Collection, stored and authenticated according to supplier’s instructions. MCF-7 cells were cultured in DMEM medium, containing 10% FBS, 1% L-glutamine and 1 mg/mL penicillin-streptomycin at 37 °C with 5% CO_2_ air. Long-term estrogen deprived (LTED) cells were derived from MCF-7 cells after estrogen-deprivation for six months [33]. MCF-7 LTED cells were grown in DMEM medium supplemented with 10% Dextran-Coated Charcoal (Sigma-Aldrich, Milano, Italy), 1% L-glutamine and 1 mg/mL penicillin-streptomycin. All cell lines, were regularly tested for morphology, doubling times, estrogen sensitivity and mycoplasma-negativity (MycoAlert Mycoplasma Detection Assay, Lonza, Basilea, CH, Switzerland).

### 4.3. Isolation of Tumor-Derived Exosomes

MCF-7 and MCF-7 LTED cells were plated at a density of 3.5 × 10^6^ cells/75 cm^2^ flask in 10 mL of complete medium for 24 h and then incubated in serum-free medium. At least 5 flasks/conditions were used. After 48 h, conditioned medium was harvested and exosomes were isolated by differential ultracentrifugation method [34]. Briefly, the first step was designed to eliminate large dead cells and cell debris by successive centrifugations at increasing speed (300× *g* and 2000× *g* for 10 min, respectively). At each of these steps, the pellet was thrown away, and the supernatant was used to following step. The resulting supernatant was centrifuged at 10,000× *g* for 30 min to remove microvesicles and the final supernatant was then ultracentrifuged at 100,000× *g* for 70 min (Sorvall WX Ultra Series Centrifuge, T-865, Thermo Fisher Scientific, Milan, Italy). The obtained pellet, that corresponds to exosome, was washed in a large volume of PBS (5 mL) to eliminate any contaminating proteins and ultracentrifuged one last time at the same high speed. All steps were carried out at 4 °C. The final exosome pellet was resuspended in PBS and stored at −80 °C until use [55].

### 4.4. Transmission Electron Microscopy (TEM)

Whole exosome extracts were fixed in 2% glutaraldehyde and then absorbed onto formovar-coated grids for 20 min in a dry environment. The grids were examined in a Jeol JEM 1400 Plus electron microscope (JEOL USA, Inc., MA, USA) at 80 kV.

### 4.5. Immunoblot Analysis

Cells and exosomes were lysed in RIPA Buffer (50 mM Tris-HCl, 150 mM NaCl, 1% Nonidet P-40, 0.5% sodium deoxycholate, 2 mM sodium fluoride, 2 mM EDTA, and 0.1% SDS) containing a mixture of protease inhibitors (aprotinin, phenylmethylsulfonyl fluoride, and sodium orthovanadate) for protein extraction. Equal amounts of cell and exosome extracts were resolved by SDS-PAGE as described in [56,57]. Images were acquired by Odissey FC (Licor, Lincoln, NE, USA) and the bands of interest were quantified using Scion Image laser densitometry scanning program (National Institutes of Health, MD, USA).

### 4.6. Nanoparticle Tracking Analysis (NTA)

Exosomes from MCF-7 and MCF-7 LTED cells were diluted in PBS before Nanoparticle Tracking Analysis (NTA), which measures the concentration and the size distribution of exosome in the 10 nm to 2 μm range. NTA was undertaken using the NanoSight NS300 technology (Malvern Panalytical Ltd., Malvern, UK) equipped with a 488 nm laser that allows the tracking of both light scattering and Brownian motion of nanoparticles in a liquid suspension on a particle-by-particle basis. The assay was performed according to the recommendation of the instrument’s manufacturer. Since NTA is more accurate between particle concentrations in the range of 2 × 10^8^ to 2 × 10^9^, esosomes from MCF-7 and MCF-7 LTED cells were diluted before analysis at 1:200 and 1:800, respectively. Briefly, sixty-second videos were recorded in five replicates for sample with optimized set parameters (the detection threshold was set to 5 for both samples). Data capture and further analysis were performed using the NTA software version 3.3 (Malvern Panalytical, LTD., Malvern, UK). Size distribution and concentration profiles were averaged across replicates to derive the presented results.

### 4.7. RNA Extraction and Real-Time RT-PCR Assays

Total RNA was extracted from exosomes using Total Exosome RNA and Protein Isolation Kit following the manufacturer’s instructions (Thermo Fisher Scientific, Milan, Italy). Total cellular RNA was extracted using TRIZOL reagent (Life Technologies, Milan, Italy) as suggested by the manufacturer. Rab gene levels were measured by real-time RT-PCR, using SYBR Green Universal PCR Master Mix (Bio-Rad, Segrate, Italy) as previously described [58]. mRNA expression levels of genes were normalized on 18s mRNA content, and relative gene expression levels were calculated as described [59]. Primers are listed in Supplementary Table S1.

### 4.8. TUNEL Assay

Apoptosis was determined by enzymatic labeling of DNA strand breaks using terminal deoxynucleotidyl transferase-mediated deoxyuridine triphosphate nick end labeling, using APO-BrdUTM TUNEL Assay Kit (Promega, Madison, WI, USA) as described [60].

### 4.9. Proteomic Analysis

#### 4.9.1. Protein Digestion for MS Analysis

For label-free quantitative proteomic analysis, MCF-7 and MCF-7 LTED cells were lysed with UA buffer (100 mM Tris HCl, pH 8.5, and 8 M urea) and the total concentration of proteins in solution was measured by Bicinchoninic acid (BCA, Thermo Fisher Scientific, Milan, Italy) assay. Next, 50 µg of lysate was in-solution digested [61]. Briefly, proteins were reduced by TCEP, alkylated by chloroacetamide, and digested by Lys-C and trypsin, then peptides were desalted on StageTip C18 [62].

#### 4.9.2. Mass Spectrometry Analysis

Each sample was analyzed as technical duplicate on a LC–ESI–MS-MS quadrupole Orbitrap QExactive-HF mass spectrometer (Thermo Fisher Scientific, Milan, Italy). Separation of peptides was achieved on a linear gradient from 93% solvent A (2% ACN, 0.1% formic acid) to 60% solvent B (80% acetonitrile, 0.1% formic acid) over 110 min, and from 60% to 100% solvent B in 10 min at a constant flow rate of 0.25 µL/min on UHPLC Easy-nLC 1000 (Thermo Fischer Scientific, Milan, Italy) connected to a 23 cm fused-silica emitter of 75 µm ID (New Objective, Inc. Woburn, MA, USA), packed in-house with ReproSil-Pur C18-AQ 1.9 µm beads (Dr Maisch Gmbh, Ammerbuch, Germany) using a high-pressure bomb loader (Proxeon, Odense, Denmark). MS data were acquired using a data-dependent top 20 method for HCD fragmentation. Survey full scan MS spectra (300–1650 Th) were acquired in the Orbitrap with resolution 60,000, AGC target 3e6, IT 20 ms. For HCD spectra, resolution was set to 15,000 at *m*/*z* 200, AGC target 1e5, IT 80 ms; NCE 28%, dynamic exclusion 20 s and isolation width 1.2 *m*/*z* [63].

#### 4.9.3. MS Analysis

Raw files were processed via MaxQuant software 1.5.2.8 (Computational Systems Biochemistry Martinsried, Germany) [64], with Andromeda search engine [65]. MS/MS peak lists were searched against the database Uniprot_cp_Human, setting trypsin specificity and up to two missed cleavages; cysteine carbamidomethyl as fixed modification, methionine oxidation and protein N-terminal acetylation as variable modifications. Mass deviation for MS-MS peaks was set at 20 ppm. The peptides and protein FDR were set to 0.01; the minimal length required for a peptide was six amino acids; a minimum of two peptides and at least one unique peptide were required for high-confidence protein identification. Proteins were analyzed in a label-free manner, using protein intensity values normalized across the entire data set. Each protein was assigned to the functional classification based on the Gene Ontology annotation system using the Database for Annotation, Visualization and Integrated Discovery version 6.8 (https://david.ncifcrf.gov/). The mass spectrometry proteomic data have been deposited to the ProteomeXchange Consortium via PRIDE partner repository with the dataset identifier PXD012431.

### 4.10. Statistical Analysis

Data were analyzed for statistical significance using a two-tailed Student’s Test and GraphPad-Prism 7 (GraphPad Inc., CA, USA). Standard deviations/S.D. are shown. Statistical analysis for proteomic analysis was performed via Perseus platform version 1.5.1.6 (Computational Systems Biochemistry, Martinsried, Germany) on Normalized Intensities by *z*-score normalization, using *t*-test, Permutation test and FDR 0.05; statistically significant proteins were submitted to Hierarchical Clustering analysis and represented on HeatMaps. In the gene ontology analysis, only categories containing at least three genes and those having a Benjamini-corrected *p* value below 0.05 were accepted as significant.

## 5. Conclusions

Evidence for the functions and roles of exosomes as mediators of therapy failure is growing, and recent studies have detected more exosome-releasing properties in drug-resistant settings [66]. To our knowledge, this is the first study reporting that the AI^R^ phenotype might be associated with an increased capability of breast cancer cells to release exosomes, raising the need to better evaluate the impact of exosomes on endocrine therapy. Therefore, based on our data, it will be intriguing and worthwhile to further investigate this mechanism in future studies aimed at clarifying: (i) which Rab GTPase may be primarily involved in exosome-mediated AI^R^ in breast cancer; (ii) which specific molecules delivered by exosomes may cause extrinsic therapy resistance; (iii) which recipient cells, such as non-resistant cancer cells, macrophages, fibroblasts, etc., can be driven by exosomal molecules from resistant cells within the “AI^R^ microenvironment”. Unraveling these events implies several clinical implications prospectively. First, because exosomes contain proteins, RNA, and many types of miRNAs whose levels can be measured in blood, urine, or other bodily fluids, their evaluation might offer new predictive markers for hormonal response. Second, a deeper understanding of the key molecules involved in AI^R^ vesiculation may help to identify potential therapeutic targets, which may be useful to extend the duration of sensitivity to estrogen deprivation, or to overwhelm resistance at its time of emergence in breast cancer patients.

## Figures and Tables

**Table 1:** Top 10 biological processes identified by proteomic analysis in MCF-7 LTED cells compared to MCF-7 cells.

Term	Count	%	*p* Value	Fold Enrichment	FDR
RNA processing	188	7.75	1.80 × 10^−31^	2.33	3.35 × 10^−28^
Establishment of protein localization	235	9.69	1.47 × 10^−30^	2.07	2.73 × 10^−27^
Protein transport	233	9.60	2.45 × 10^−30^	2.07	4.56 × 10^−27^
Translation	133	5.48	8.43 × 10^−30^	2.72	1.57 × 10^−26^
Protein localization	255	10.51	4.90 × 10^−29^	1.96	9.13 × 10^−26^
RNA splicing	118	4.86	5.10 × 10^−28^	2.81	9.49 × 10^−25^
mRNA metabolic processing	138	5.69	4.41 × 10^−27^	2.53	8.20 × 10^−24^
Intracellular transport	203	8.38	4.87 × 10^−27^	2.09	9.07 × 10^−24^
mRNA processing	123	5.07	2.14 × 10^−25^	2.60	3.98 × 10^−22^
Vesicle-mediated transport	168	6.92	1.39 × 10^−19^	1.98	2.59 × 10^−16^

Differentially expressed proteins, determined by proteomic analysis, were analyzed to outline the most enriched biological processes. The table shows the top ten enriched terms that correspond to biological processes, along with the count of proteins involved, *p* value, fold enrichment and False Discovery Rate (FDR).

**Table 2:** Selected up-regulated proteins identified by proteomic analysis in MCF-7 LTED cells compared to MCF-7 cells (FDR < 0.05).

Protein ID	Protein Name	Gene Names	Ratio AVG
Q969Q5	Ras-related protein Rab-24	*RAB24*	6.41074493
Q6WKZ4	Rab11 family-interacting protein 1	*RAB11FIP1*	3.283109519
Q13637	Ras-related protein Rab-32	*RAB32*	3.134080381
A0A024R2K1	Ras-related protein Rab-5A	*RAB5A*	2.684524433
Q8WUD1	Ras-related protein Rab-2B	*RAB2B*	2.358702001
A0A024RD41	Ras-related protein Rab-23	*RAB23*	1.874367334
Q5HYI8	Rab-like protein 3	*RABL3*	1.832400186
Q9UL26	Ras-related protein Rab-22A	*RAB22A*	1.72827705
Q6IQ22	Ras-related protein Rab-12	*RAB12*	1.608127936
A0A024RBA9	Ras-related protein Rab-21	*RAB21*	1.583370444
A0A024R5J5	Ras-related protein Rab-6A	*RAB6A*	1.470976838
A0A024R7V6	Ras-related protein Rab-2A	*RAB2*; *RAB2A*	1.455072096
Q9NP72	Ras-related protein Rab-18	*RAB18*	1.451684135
A0A024R7G2	Ras-related protein Rab-3D	*RAB3D*	1.42786787
A0A024R7I7	Ras-related protein Rab-3A	*RAB3A*	1.41943675
Q15907	Ras-related protein Rab-11B; Ras-related protein Rab-11A	*RAB11B*; *RAB11A*	1.403714286
A0A024R845	Ras-related protein Rab-14	*RAB14*	1.381289829
A0A024RB09	Ras-related protein Rab-5B	*RAB5B*	1.333981668
A0A158RFU6	Ras-related protein Rab-7a	*RAB7A*	1.332295086
O00194	Ras-related protein Rab-27B	*RAB27B*	1.327797939
A0A024R1U4	Ras-related protein Rab-5C	*RAB5C*	1.307181423

The table shows differentially expressed proteins in MCF-7 and MCF-7 LTED cells, identified by proteomic analysis, along with the names of the corresponding gene and the ratio average between MCF-7 LTED and MCF-7 cells.

## Supplementary Materials

Supplementary materials can be found at https://www.mdpi.com/1422-0067/21/16/5841/s1, Figure S1: Evaluation of apoptosis in MCF-7 and MCF-7 LTED cells, Table S1: Oligonucleotide primers used in this study.
