# Supplementary material for: Evidence for Enhanced Exosome Production in Aromatase Inhibitor-Resistant Breast Cancer Cells
Source: Int J Mol Sci. 2020 Aug 14;21(16):5841. doi: 10.3390/ijms21165841 (PMC7461508; doi:10.3390/ijms21165841)
Supplement: Supplementary file 1 [file ijms-21-05841-s001.pdf]

Supplementary Materials

Evidence for Enhanced Exosome Production in Aromatase Inhibitor-Resistant Breast Cancer Cells

Giuseppina Augimeri [1,†], Giusi La Camera [1,†], Luca Gelsomino [1], Cinzia Giordano [1,2], Salvatore Panza [1], Diego Sisci [1], Catia Morelli [1], Balázs Győrffy [3,4], Daniela Bonofiglio [1,2], Sebastiano Andò [1,2,‡], Ines Barone [1,*,‡] and Stefania Catalano [1,2,*,‡]

[1] Department of Pharmacy, Health and Nutritional Sciences, Via P Bucci, University of Calabria, 87036 Arcavacata di Rende (CS), Italy; giusy.augimeri@gmail.com (G.A.); giusylacamera93@gmail.com (G.L.C.); luca.gelsomino@unical.it (L.G.); cinzia.giordano@unical.it (C.G.); sasapanza@libero.it (S.P.); dsisci@unical.it (D.S.); catia.morelli@unical.it (C.M.); daniela.bonofiglio@unical.it (D.B.)

[2] Centro Sanitario, Via P Bucci, University of Calabria, 87036 Arcavacata di Rende (CS), Italy

[3] Bioinformatics and 2nd Department of Pediatrics, Semmelweis University, 1094 Budapest, Hungary; zsalab2@yahoo.com (B.G.)

[4] TTK Cancer Biomarker Research Group, 1117 Budapest, Hungary

* Correspondence: ines.barone@unical.it (I.B.); stefania.catalano@unical.it (S.C.); Tel.: +39-0984-496216 (I.B.); +39-0984-496207 (S.C.)

† These authors contributed equally to this work.

‡ Joint senior author.

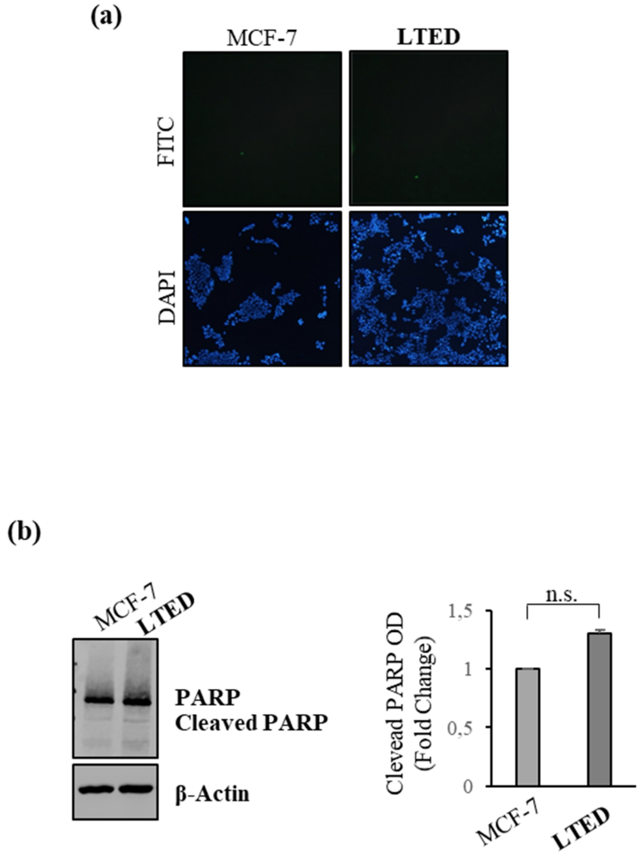

Figure S1. Evaluation of apoptosis in MCF-7 and MCF-7 LTED cells. (**a**) Terminal deoxynucleotidyk transferase-mediated dUTP nick end labeling (TUNEL) staining in MCF-7 and LTED cells in serum-free medium for 48h. DAPI was used for nuclear staining; (**b**) Immunoblot analysis of PARP and cleaved-PARP protein levels from total MCF-7 and LTED cellular extracts. β-Actin was used as a control for equal loading and transfer. The histograms represent the mean ± SD of three separate experiments in which band intensities were evaluated in terms of optical density arbitrary units (OD) and expressed as fold over MCF-7 cells. n.s., nonsignificant.

Table S1. Oligonucleotide primers used in this study.

Gene Symbol	Gene Name	Primer Sequences
RAB3D	Member RAS oncogene family RAB3D	For 5′- TCAAGACCGTCTACCGCCAT -3′<br>Rev 5′- CTTGATTTGCGTGGCCCAGTC -3′
RAB5A	Member RAS oncogene family RAB5A	For 5′- CTAGTCGAGGCGCAACAAGA -3′<br>Rev 5′- GGGTTAGAAAAGCAGCCCCA -3′
RAB5B	Member RAS oncogene family RAB5B	For 5′- CGGAGCCCCAGGAGTGTT -3′<br>Rev 5′- CACTGCAGATTCTCCCAGCA -3′
RAB6A	Member RAS oncogene family RAB6A	For 5′- GCCCGTGCAAATGTATCTGTG -3′<br>Rev 5′- GACCCGCAGTATCCCACAG -3′
RAB7A	Member RAS oncogene family RAB7A	For 5′- CCTCGAAAACAGACAAGTGGC -3′<br>Rev 5′- ATTCCGTGCAATCGTCTGGA -3′
RAB11A	Member RAS oncogene family RAB11A	For 5′- ACGTCATCTCAGGGCAGTTC-3′<br>Rev 5′- GAGAAACAATGCGGTAAATCTCTGT -3′
RAB12	Member RAS oncogene family RAB12	For 5′- TCATCATTATCGGCTCCCGC -3′<br>Rev 5′- TTGAAGTCAACACCCACGGT -3′
RAB14	Member RAS oncogene family RAB14	For 5′- ACCATGGCAACTGCACCATA -3′<br>Rev 5′- TCCTACTCCCATGTCCCCAAT -3′
RAB18	Member RAS oncogene family RAB18	For 5′- ACTTGCAGCAACAATAGGTGT -3′<br>Rev 5′- TAACACCCTGTGCACCTCTA -3′
RAB21A	Member RAS oncogene family RAB21A	For 5′- AACGACAAGCACATCACCAC -3′<br>Rev 5′- TCTTGACCTGCCGTATCCCAT -3′
RAB22A	Member RAS oncogene family RAB22A	For 5′- TCAACCCAACAATAGGGGCA -3′<br>Rev 5′- ATTATAGCTGCAGCCGACCC -3′
RAB23	Member RAS oncogene family RAB23	For 5′- TCCCGGGTTGTGCCTTACT -3′<br>Rev 5′- TCTTCCCTCTCAAATCGCTGGG-3′
RAB24	Member RAS oncogene family RAB24	For 5′- GAGATCGGGGTTTGCCTCCC -3′<br>Rev 5′- CGGCCCCGATGGTGTTCT -3′
RAB27B	Member RAS oncogene family RAB27B	For 5′- ATAAGTAGCTGTCCCCGTGC -3′<br>Rev 5′- GATGGTCTTGGTCGGTCAGC -3′
RAB32	Member RAS oncogene family RAB32	For 5′- CATCGCGGGGCAGGAG -3′<br>Rev 5′- AGAGGACAGCAGGGATAGGG -3′
